# Plasma Membrane Profiling Defines an Expanded Class of Cell Surface Proteins Selectively Targeted for Degradation by HCMV US2 in Cooperation with UL141

**DOI:** 10.1371/journal.ppat.1004811

**Published:** 2015-04-14

**Authors:** Jye-Lin Hsu, Dick J. H. van den Boomen, Peter Tomasec, Michael P. Weekes, Robin Antrobus, Richard J. Stanton, Eva Ruckova, Daniel Sugrue, Gavin S. Wilkie, Andrew J. Davison, Gavin W. G. Wilkinson, Paul J. Lehner

**Affiliations:** 1 Cambridge Institute for Medical Research, University of Cambridge, Cambridge, United Kingdom; 2 School of Medicine, Cardiff University, Cardiff, United Kingdom; 3 Regional Centre for Applied Molecular Oncology (RECAMO), Masaryk Memorial Cancer Institute, Brno, Czech Republic; 4 MRC-University of Glasgow Centre for Virus Research, University of Glasgow, Glasgow, United Kingdom; University Medical Center, Albert-Ludwigs-University Freiburg, GERMANY

## Abstract

Human cytomegalovirus (HCMV) US2, US3, US6 and US11 act in concert to prevent immune recognition of virally infected cells by CD8+ T-lymphocytes through downregulation of MHC class I molecules (MHC-I). Here we show that US2 function goes far beyond MHC-I degradation. A systematic proteomic study using Plasma Membrane Profiling revealed US2 was unique in downregulating additional cellular targets, including: five distinct integrin α-chains, CD112, the interleukin-12 receptor, PTPRJ and thrombomodulin. US2 recruited the cellular E3 ligase TRC8 to direct the proteasomal degradation of all its targets, reminiscent of its degradation of MHC-I. Whereas integrin α-chains were selectively degraded, their integrin β1 binding partner accumulated in the ER. Consequently integrin signaling, cell adhesion and migration were strongly suppressed. US2 was necessary and sufficient for degradation of the majority of its substrates, but remarkably, the HCMV NK cell evasion function UL141 requisitioned US2 to enhance downregulation of the NK cell ligand CD112. UL141 retained CD112 in the ER from where US2 promoted its TRC8-dependent retrotranslocation and degradation. These findings redefine US2 as a multifunctional degradation hub which, through recruitment of the cellular E3 ligase TRC8, modulates diverse immune pathways involved in antigen presentation, NK cell activation, migration and coagulation; and highlight US2’s impact on HCMV pathogenesis.

## Author Summary

As the largest human herpesvirus, HCMV is a paradigm of viral immune evasion and has evolved multiple mechanisms to evade immune detection and enable survival. The HCMV genes US2, US3, US6 and US11 promote virus persistence by their ability to downregulate cell surface MHC. We developed ‘Plasma Membrane Profiling’ (PMP), an unbiased SILAC-based proteomics technique to ask whether MHC molecules are the only focus of these genes, or whether additional cellular immunoreceptors are also targeted. PMP compares the relative abundance of cell surface receptors between control and viral gene expressing cells. We found that whereas US3, US6 and US11 were remarkably MHC specific, US2 modulated expression of a wide variety of cell surface immunoreceptors. US2-mediated proteasomal degradation of integrin α-chains blocked integrin signaling and suppressed cell adhesion and migration. All US2 substrates were degraded via the cellular E3 ligase TRC8, and in a remarkable example of cooperativity between HCMV immune-evasins, UL141 requisitioned US2 to target the NK cell ligand CD112 for proteasomal degradation. HCMV US2 and UL141 are therefore modulators of multiple immune-related pathways and act as a multifunctional degradation hub that inhibits the migration, immune recognition and killing of HCMV-infected cells.

## Introduction

HCMV is the prototype betaherpesvirus and an important human pathogen. Following primary infection, HCMV persists for the lifetime of the host under constant control by the host immune system. In the face of this selective pressure, HCMV has evolved multiple mechanisms to evade immune detection and has emerged as a paradigm of viral immune modulation and evasion. Experimentally, only 45 of the ~170 canonical HCMV protein coding genes are required for *in vitro* replication [1, 2]; most HCMV genes appear to be directed at promoting virus persistence through targeting host defenses [3–5].

Four genes clustered in the HCMV unique short (US) gene region use independent mechanisms to suppress MHC-I dependent antigen presentation to CD8+ cytotoxic T lymphocytes [6]. US3 is an immediate early gene product that binds and retains newly synthesized MHC-I proteins in the endoplasmic reticulum (ER) and blocks tapasin-dependent peptide loading [7, 8], whereas US6 inhibits TAP-mediated peptide translocation into the ER [9, 10]. US2 and US11 both bind MHC-I in the lumen of the ER and hijack the mammalian ER-associated degradation (ERAD) machinery to promote retrotranslocation to the cytosol for proteasome degradation [11, 12]. US2 and US11 appropriate distinct cellular ERAD pathways for MHC I dislocation. US2 utilizes the cellular E3 ligase TRC8 (translocation in renal cancer from chromosome 8) to ubiquitinate and subsequently degrade MHC-I [13], whereas US11 uses a Derlin-1-associated ERAD complex centered around the newly characterized TMEM129 E3 ligase [14–16]. Functionally US2 and US11 are distinct as US11 has a combined ER retention and degradation function [13, 15, 17], while US2 is unable to retain MHC-I in the ER prior to degradation, but relies on US3 for enhanced degradation. In addition to downregulating MHC-I, US2 and US3 also target the MHC-II antigen presentation pathway [18, 19]. US3 retains MHC-II molecules in the ER while US2 initiates the retrotranslocation of MHC-II DR-α chain and DM-α chain from the ER back to the cytosol for subsequent degradation.

Since endogenous MHC-I molecules constitute the chief ligands recognized by NK cell inhibitory receptors, their downregulation has the potential to render cells more vulnerable to NK cell attack. To compensate, HCMV encodes its own MHC-I homologue (UL18) and a peptide present in the UL40 signal sequence acts to stabilize and maintain cell surface expression of the NK inhibitory ligand HLA-E [20–24]. Moreover, HCMV systematically suppresses cell surface expression of ligands for NK cell activating receptors. The HCMV glycoprotein UL141 plays a major role in such protection via interaction with TRAIL death receptors, as well as CD155 (PVR, necl5) and CD112 (PVRL2, nectin-2) which are both ligands for the ubiquitous NK activating receptor DNAM1 [25–27]. In isolation, UL141 is capable of suppressing both CD155 and TRAIL-R2 cell surface expression, but an additional HCMV-encoded factor is known to be required for efficient downregulation of cell surface CD112 [25]. Moreover, while CD155 and TRAIL-R2 accumulate in the ER during the course of infection, CD112 is degraded [25].

While US2, US3, US6 and US11 were originally defined by their capacity to inhibit cell surface MHC-I expression, classical MHC molecules are not necessarily their only targets. To gain an unbiased view of cellular receptors whose expression is altered upon viral gene expression, we recently developed ‘Plasma Membrane Profiling’ (PMP), a SILAC (Stable Isotope Labelling of Amino acids in Culture)-based quantitative proteomics technique which compares the relative abundance of cell surface receptors between infected and uninfected cells, and therefore identifies the range of cell surface proteins downregulated upon viral infection [28–30]. PMP demonstrated that whereas US3, US6 and US11 specifically downregulate MHC-I, US2 targets a series of novel substrates including the NK cell ligand CD112, the anti-coagulation factor thrombomodulin and at least six integrin family members, abolishing integrin signalling, cell adhesion and migration. While US2 alone is necessary and sufficient to target most substrates, effective downregulation of CD112 requires a synergistic interaction between US2 and UL141. UL141 retains CD112 in the ER and associates with US2 resulting in CD112 dislocation across the ER membrane for proteasome degradation. We therefore propose a role for US2 that is much broader than previously appreciated, but nevertheless depends on the common activity of TRC8 for impact. Furthermore, US2 and UL141 form a multifunctional and highly adaptive degradation hub with a substrate range much wider than previously appreciated, affecting cellular processes as broad as antigen presentation, NK cell killing, cell migration and coagulation.

## Results

### Plasma membrane profiling identifies novel HCMV US2 specific substrates

**Fig 1:**
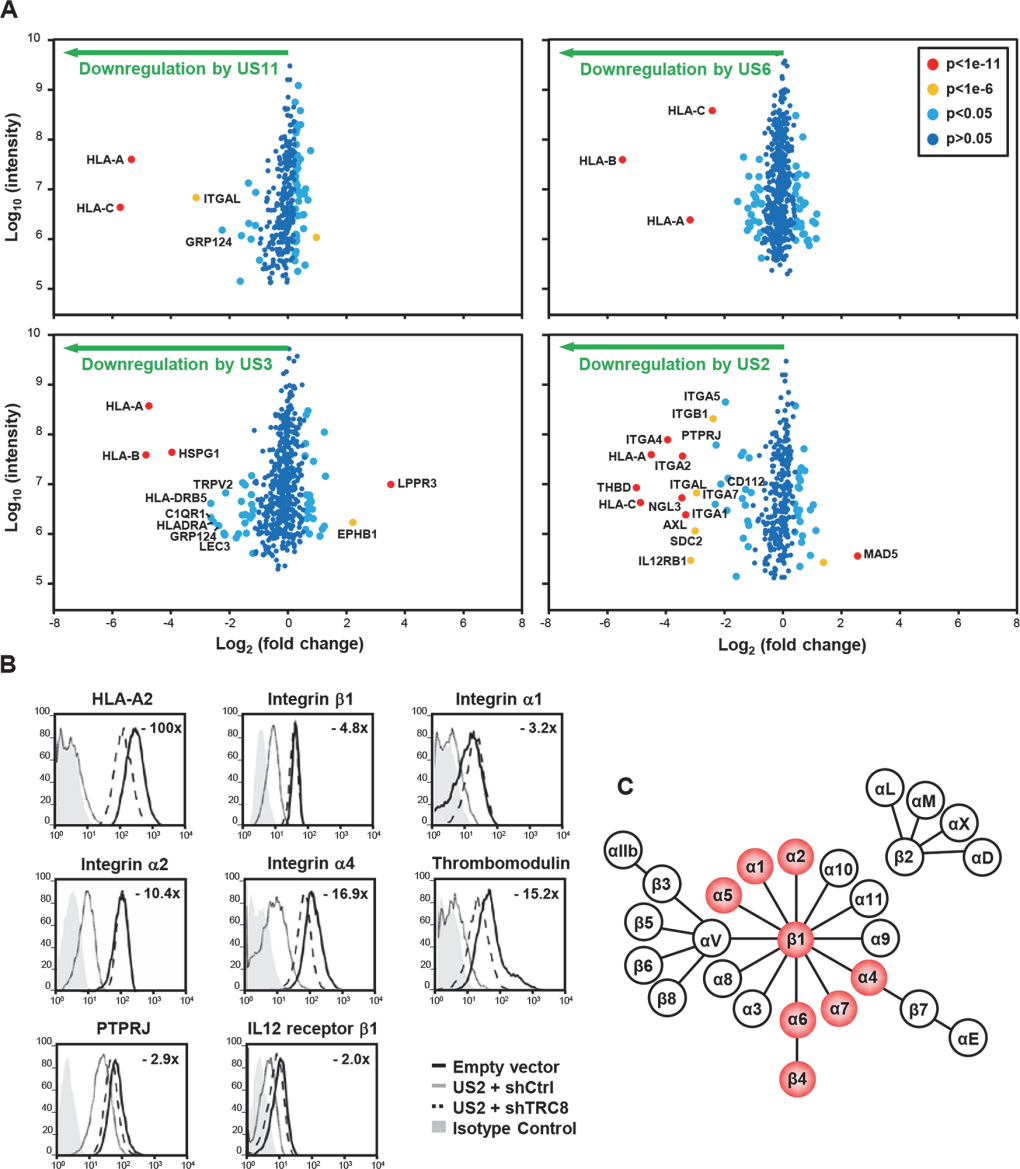
Identification of novel US2 specific substrates by plasma membrane profiling. US2 downregulates cell surface expression of α and β integrins in addition to other proteins. (**A**) Scatter plots of proteins identified in PMP and quantified by 2 or more unique peptides. Fold change (single gene expressing THP-1 cells / control THP-1 cells) is shown as log_2_ ratio on the x axis and the summed peptide intensity on the y-axis as log_10_. Proteins unaltered by viral gene expression locate to the center of the plots (0-fold change), whereas proteins left or right of center represent respectively proteins down- or up-regulated by viral gene expression. Significance B was used to estimate p values. (**B**) Verification of integrin downregulation in US2-expressing THP-1. Cytofluorometric analysis of the indicated proteins in THP-1 cells stably expressing US2 with or without TRC8 depletion, and control THP-1 cells. (**C**) Representation of the integrin family. Integrins downregulated by US2 are highlighted in red. See also **S1 and S2 Figs** and **S1 Table**.

To determine whether the HCMV-encoded US2, 3, 6 and 11 viral gene products downregulate cell surface proteins in addition to MHC molecules, we used Plasma Membrane Profiling (PMP), an unbiased, proteomic technique which compares the relative abundance of plasma membrane proteins [28]. Plasma membrane proteins were isolated from THP-1 monocytic cells stably expressing HCMV US2, US3, US6 or US11 by sequential cell surface glycoprotein biotinylation followed by streptavidin pull-down and their relative expression was quantified by high through-put mass spectrometry. To minimise differences in sample preparation, cells were metabolically labelled prior to biotinylation by SILAC, which allows early stage sample mixing without loss of sample identity [31]. The relative abundance of plasma membrane proteins in US2, US3, US6 or US11 expressing cells versus control is plotted in **Fig 1A** with individual proteins represented by single dots. Those proteins whose expression is unaltered by viral gene expression accumulate in the centre, whereas left and right shifts represent proteins down- or up-regulated at the plasma membrane of viral gene expressing cells.

In US3, US6 and US11 expressing cells, the majority of plasma membrane proteins identified (370–454) were unchanged, with HLA-A, B and C allotypes of MHC-I molecules being the predominant proteins lost from the cell surface (**Fig 1A;** left and top right panels). US3 also showed a decrease in MHC-II and C1q complement receptor expression. In contrast, US2 altered the expression of a multitude of cell surface receptors, with thirteen new proteins showing more than a four-fold downregulation (**Fig 1A** bottom right panel, **S1 Table**). In addition to MHC-I, six integrin family members were downregulated: α1 (ITGA1, 4.9 fold), α2 (ITGA2, 10.7 fold), α4 (ITGA4, 15.3 fold), α5 (ITGA5, 3.9 fold), α7 (ITGA7, 4.3 fold) and β1 (ITGB1, 5.2 fold). Other substrates downregulated by US2 include thrombomodulin (THBD, 31.9 fold), protein tyrosine phosphatase, receptor type, J (PTPRJ, 4.9 fold) and the interleukin-12 receptor β1 (IL12RB1, 8.9 fold).

### US2 substrates are downregulated in a TRC8-dependent pathway

We focused on novel US2 substrates and confirmed their downregulation by flow cytometry. Indeed US2 expressing THP-1 cells showed a robust downregulation of integrins α1, α2, α4, β1, thrombomodulin, PTPRJ and IL12 receptor β1 (**Fig 1B, grey line**), compared to control (**black line**). Other cell surface molecules, including the transferrin receptor and integrin αV, remained unaffected by US2 and none of the US2 substrates were affected by US11 expression (**S1 Fig**), thus confirming the specificity of substrate down-regulation. AXL, integrin αM (ITGAM) and αL (ITGAL) expression was dysregulated by lentiviral transduction and not followed further.

US2 is a type I membrane protein that co-opts the cellular ERAD degradation machinery to degrade MHC-I [12]. The ER-resident ubiquitin E3 ligase TRC8 is a critical component of the US2-mediated MHC-I degradation pathway [13]. US2 recruitment of TRC8 is required for the ubiquitination and subsequent degradation of newly synthesized MHC-I, and in the absence of this ligase MHC-I is rescued back to the cell surface [13]. In a similar manner, shRNA knock-down of TRC8 in US2-expressing THP-1 cells rescued cell surface expression of integrins, THBD, PTPRJ and IL-12Rβ1 (**Figs** 1**B** **dashed line and S2**). The recruitment of TRC8 thus appears to be a common and essential step in the US2 pathway.

### US2 induces proteasomal degradation of integrin -chains and secondary accumulation of an immature integrin β1 in the ER

**Fig 2:**
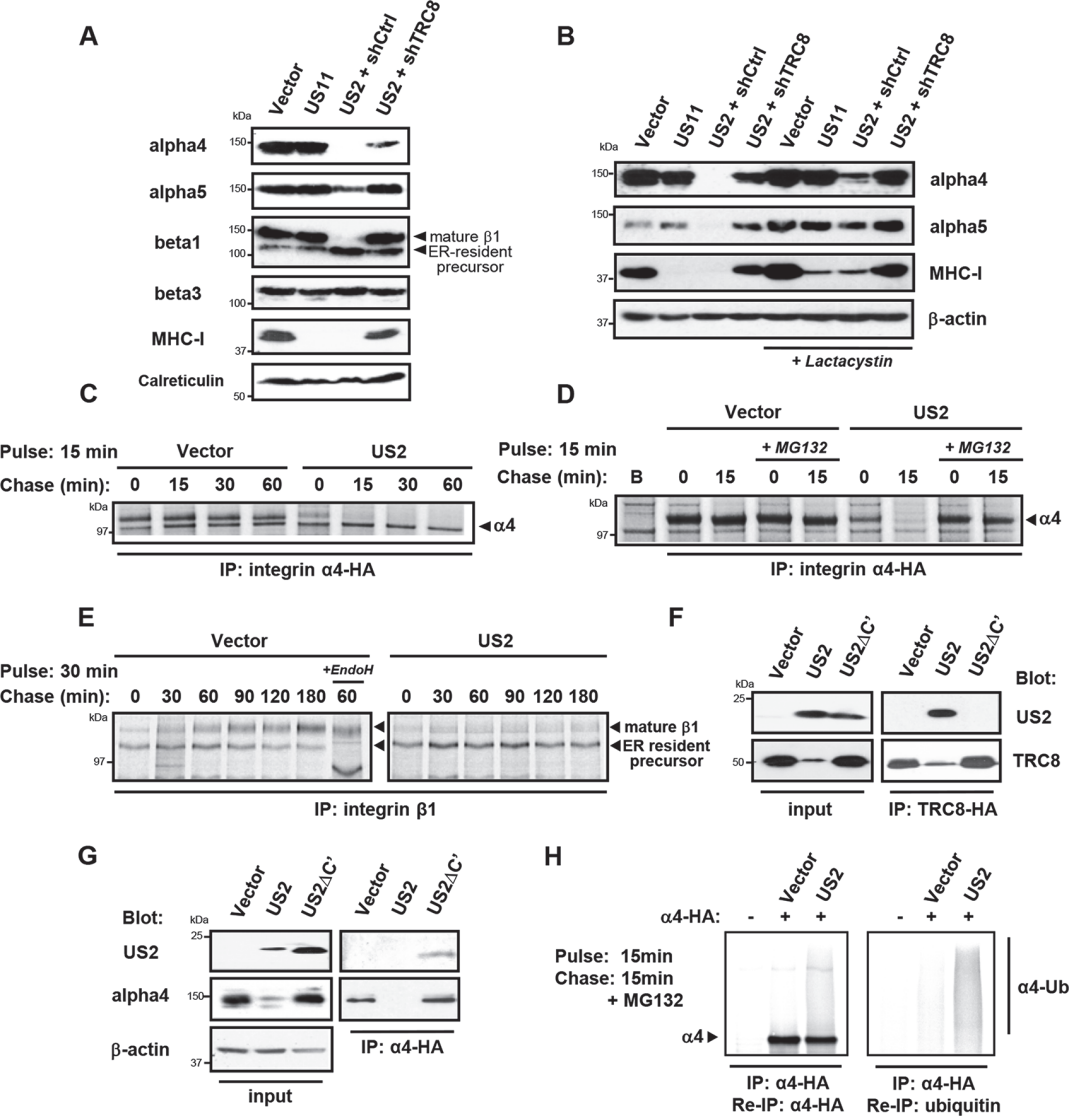
US2 induces the proteasomal degradation of integrin α4 and α5 and prevents maturation of the β1 integrin. (**A and B**) US2 induces proteasomal degradation of integrins α4 and α5. THP-1 cells stably expressing US2, US11 or depleted of TRC8 (shTRC8) were analyzed by immunoblots for the indicated proteins. Cells were treated with the proteasome inhibitor lactacystin for 18 hours before harvest as indicated. (**C-D**) Integrin 4 is proteasomally degraded in US2-expressing cells. THP-1 cells expressing integrin α4-HA were labeled with [^35^S]methionine-cysteine and pulse-chased for the indicated times in the presence or absence of MG132, followed by integrin α4 immunoprecipitation using the HA-tag **(E)** β1 integrin accumulates in its precursor form in the presence of US2. THP-1 cells were pulse-chase labelled as above and endogenous β1 integrin was immune precipitated using anti-integrin β1 antibody. (**F and G**) US2 associates with integrin α4 and recruits the TRC8 E3 ligase via its cytosolic tail. Cells stably expressing full-length US2 (US2) or US2 with Δ186–199 residues (US2ΔC') in combination with TRC8-HA or integrin α4-HA were solubilised in 1% digitonin, immunoprecipitated with anti-HA antibody and visualized by immunoblot. (**H**) US2 induces integrin α4 ubiquitination. THP-1 cells expressing integrin α4-HA in combination with US2 or empty vector were pulse-chase labeled for the indicated times. Integrin α4 was immune precipitated using the HA-epitope tag, eluted from beads, fully denatured to dissociated non-covalently linked proteins and re-precipitated using α-HA or α-ubiquitin antibodies.

To further examine the fate of these novel US2 substrates, we focused first on the integrin family. Integrins consist of an / chain heterodimer. All integrin alpha chains downregulated by US2 share the common beta-1 chain as their binding partner (**Fig 1C**). Integrins require α/β dimerisation prior to transport to the cell surface and loss of either may result in ER retention of the other subunit. It was therefore important to test whether the various α subunits or the β1 chain itself constitute the primary US2 substrate. On immunoblots integrin 4, 5 and 6 expression was strongly decreased in US2-expressing THP-1 cells (**Figs** 2**A** **lane 3 and S3**), suggesting not only down-regulation from the cell surface but US2-dependent degradation. Indeed integrin expression was rescued by proteasome inhibition (**Fig 2B lane 7**) or shRNA-mediated depletion of the TRC8 E3 ligase (**Fig 2A and 2B lane 4**). US11-expressing cells showed no change in integrin expression (**Fig 2A and 2B lane 2**). Unexpectedly, the integrin 1 chain was not itself degraded in US2-expressing cells, but accumulated in its faster migrating ER-resident immature form (**Fig 2A lane 3**). TRC8 depletion rescued this immature species to its mature form, while expression of the control integrin 3 was unaffected by US2 (**Fig 2A lanes 3 and 4**).

We used [^35^S]-methionine radiolabeling and pulse-chase analysis to further examine how US2 affects 4 and 1 integrin maturation. Integrin 4 was rapidly degraded in US2-expressing cells with a marked reduction in its half-life from more than 1 hour to less than 15 minutes (**Fig 2C**), which was prevented by the use of proteasome inhibitors (**Fig 2D**). In contrast, in the presence of US2, the 1 integrin was neither degraded, nor was it able to mature to its higher molecular species but remained in the ER in its immature form throughout the course of the 3 hour chase (**Fig 2E**). Our data suggest that α integrins are direct substrates for the US2/TRC8 pathway of proteasomal degradation, whereas ER retention of the β1 integrin is likely secondary to degradation of its α integrin interaction partners.

### The luminal domain of US2 binds integrin alpha4 and the US2 cytoplasmic tail is essential for recruiting the TRC8 E3 ligase for substrate degradation

US2 rapidly degrades its target proteins, making it difficult to ascertain whether they physically interact with US2. A truncated US2 mutant (US2ΔC'), from which the cytoplasmic tail was deleted (aa 186–199) is reported to be functionally inactive, but can still bind its MHC-I substrate [32], providing a useful tool to probe US2 interactions. We initially tested whether the US2 cytosolic domain is responsible for TRC8 recruitment, which would explain the US2ΔC' loss of function. While wild-type US2 readily binds TRC8 [13], this association is lost in the US2ΔC' mutant (**Fig 2F lanes 5 and 6**), explaining the loss of function phenotype. Furthermore, the US2ΔC' mutant is now found associated with, but unable to degrade integrin α4 (**Fig 2G lanes 3 and 6)**, suggesting that US2 binds its α integrin substrate prior to recruitment of TRC8.

Ubiquitination by TRC8 triggers the US2-induced retrotranslocation and degradation of MHC-I [13]. We therefore examined whether integrin α4 is also ubiquitinated in the presence of US2. Immune precipitation of radiolabelled integrin α4 visualized a smear of ubiquitinated species in the presence but not absence of US2 (**Fig 2H**). These species were recovered upon denaturation and re-precipitation of integrin α4 as well as ubiquitin. Integrin α4 is thus ubiquitinated in a US2-dependent manner triggering its proteasomal degradation.

### US2-mediated integrin degradation inhibits downstream integrin signalling, cell adhesion and migration

**Fig 3:**
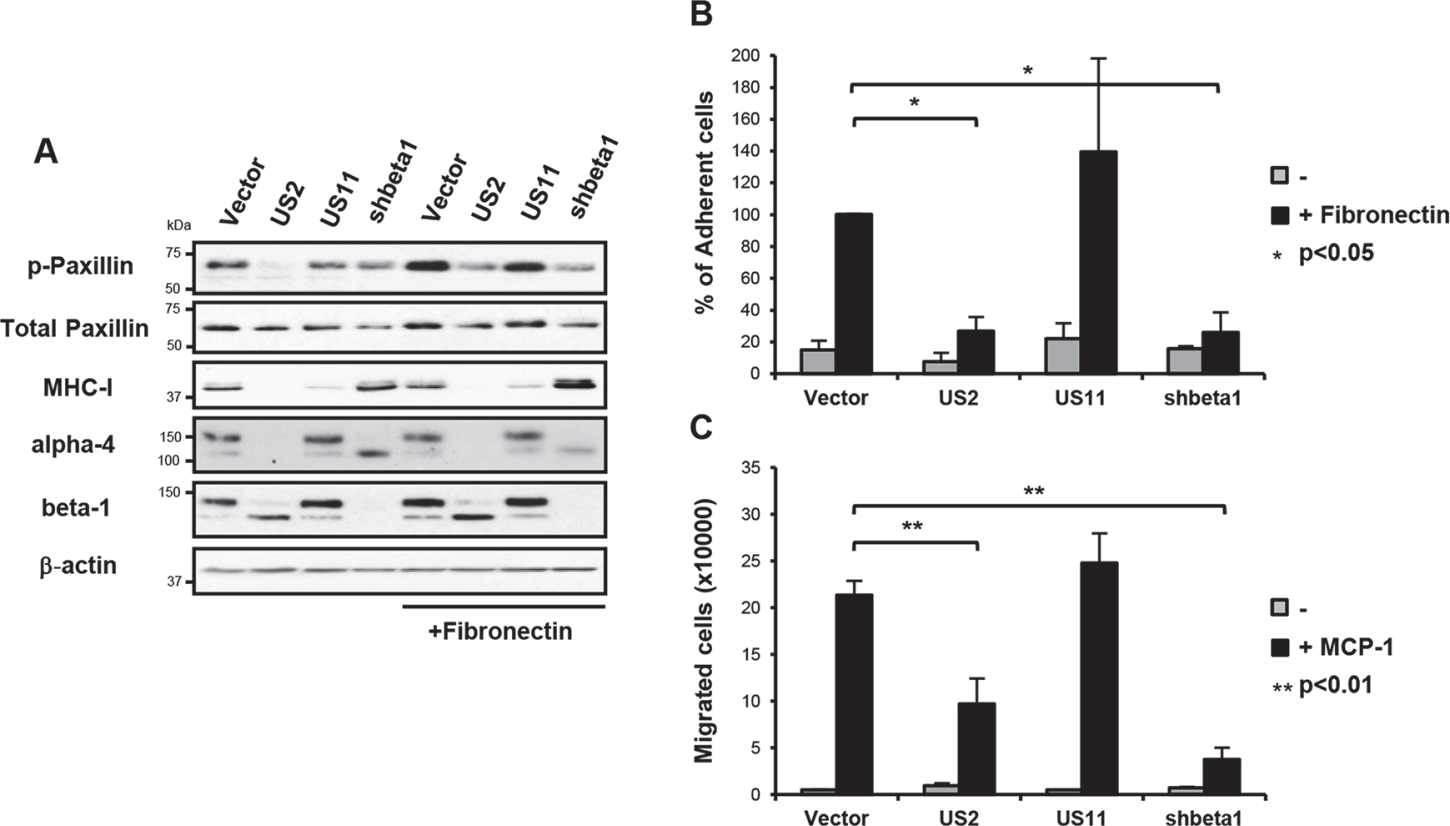
US2-mediated downregulation of integrins inhibits downstream integrin-mediated signalling, cell adhesion and migration. (**A**) US2 inhibits signaling downstream of integrin α4β1. THP-1 cells were seeded on tissue culture plates coated +/- fibronectin as indicated for 1 hour at 37°C. Total cell lysates of adherent and non-adherent cells were analyzed by immunoblotting with phospho-specific antibody to tyr118 paxillin and total paxillin. (**B**) US2 inhibits THP-1 cell adhesion. THP-1 cells were seeded in triplicate on tissue culture plates coated with fibronectin as indicated for 1 hour at 37°C and washed 3 times with PBS. Adherent cells were quantified by CyQUANT-NF (Invitrogen). The percent adherent cells was normalized against control cells expressing empty vector (vector/US2 n = 3; US11/shbeta1 n = 2). (**C**) US2 inhibits cell migration. THP-1 cell migration +/- chemotaxis agent MCP-1 (10 nM) was examined in cells stably expressing US2, US11, control or ITGB1 shRNA (shbeta1) using fibronectin-coated Transwell plates. Number of cells migrated to the lower compartment is indicated (average of triplicates, n = 4). Data are represented as mean ± SEM. p-values were calculated using paired Student’s t-test.

To address the functional consequence of US2-mediated integrin downregulation, we focused on the signaling properties of the most dramatically down-regulated integrin, α4β1. The importance of the α4β1 integrin in embryogenesis and disease pathogenesis derives from its role in cell adhesion and cell migration [33, 34]. Binding of integrin α4β1 to its ligands fibronectin or vascular cell adhesion molecule-1 (VCAM-1) initiates focal adhesion complex assembly and phosphorylation of paxillin [35, 36]. This phosphorylation event is specific to integrin β1 and α4 tails, and is not stimulated by other α-integrins [33–35]. Fibronectin stimulation of vector-only and US11-transduced control cells led to the expected increase in paxillin phosphorylation (**Fig 3A, top panel, lanes 5 and 7**), while US2 inhibited phosphorylation of paxillin as effectively as shRNA-induced depletion of the β1 integrin (**lanes 6 and 8**). Since the α4β1 integrin is required for macrophage chemotaxis, we examined how US2 affects cell adhesion and migration. Adhesion of US2-expressing THP-1 cells to a fibronectin substrate was completely abolished (**Fig 3B**) and migration of these cells was significantly reduced (**Fig 3C**) compared to the empty vector and US11 controls. A similar phenotype was observed in β1 integrin depleted cells. Thus US2-induced downregulation of integrin α4β1 has dramatic functional consequences, inhibiting downstream integrin-mediated signalling, cell adhesion and migration.

### US2 modulates cellular gene expression in productive HCMV infection

**Fig 4:**
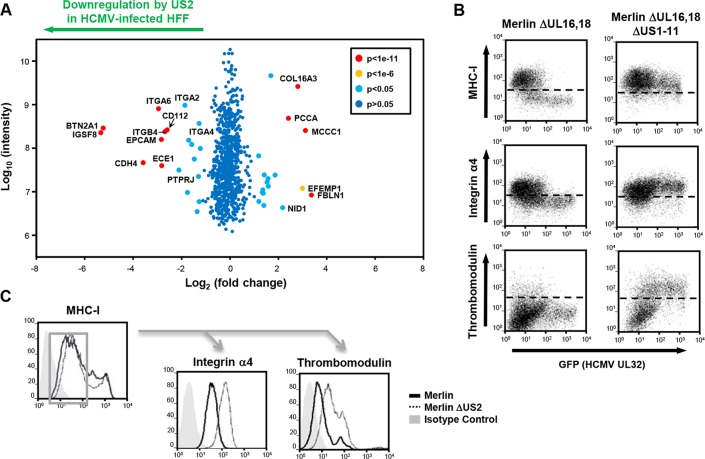
PMP comparing wild-type and US2-deficient HCMV shows a requirement for US2 in the downregulation of cell surface targets. (A) Scatter plots of proteins identified in PMP and quantified by 2 or more unique peptides. Fold change (HFF infected with wild-type HCMV versus HFF infected with ΔUS2 HCMV) is shown as log_2_ ratio on the x axis and the summed peptide intensity on the y-axis as log_10_. Proteins unaltered by HCMV US2 gene deletion locate to the center of the plots (0-fold change), whereas proteins left or right of center represent respectively proteins down- or up-regulated by HCMV in a US2-dependent manner. Significance B was used to estimate p values. (**B**) US2 is required for integrin and thrombomodulin downregulation during HCMV infection. HFF cells infected with GFP tagged HCMV ΔUL16-18 or ΔUL16-18ΔUS1-11 (moi 0.5) were analyzed by cytofluorometric analysis of the indicated proteins at 72 hours post-infection. (**C**) HFFs infected with HCMV wild-type or ΔUS2 (moi 0.5) were analyzed by flow cytometry at 72 hours post-infection. Cell surface staining for MHC-I enabled gating for HCMV infected (MHC-I^lo^) cells (left panel) and subsequent analysis of the integrin and thrombomodulin expression (right panels). See also **S3 Table**.

US2 expression alone is both necessary and sufficient for downregulation and TRC8-dependent degradation of the novel substrates. We next sought to investigate US2 function in the context of a productive HCMV infection and, in an experiment complementary to US2 single gene expression, evaluated the effect of deleting US2 from the HCMV genome using PMP. Permissive human foreskin fibroblasts (HFF) were infected with the HCMV strain Merlin (HCMV wt) or a US2 deletion virus (HCMV ΔUS2), SILAC labeled and plasma membrane proteins were isolated and quantified. Proteins whose relative abundance is higher in cells infected with HCMV ΔUS2 compared to those infected with wt-HCMV require US2 for their downregulation (left shift in **Fig 4A**). Of 686 plasma membrane proteins identified from HCMV-infected HFFs, four integrin family members (α2 (3.6 fold), α4 (3.2 fold), α6 (7.7 fold), and β4 (6.4 fold) (**Fig 4A and S2 and S3 Tables**) as well as PTPRJ were significantly downregulated in a US2-dependent manner. In addition, other immunoglobulin superfamily members that had not been identified in THP-1 cells also required US2 for their downregulation. These included immunoglobulin superfamily member 8 (IGSF8) and epithelial cell adhesion molecule (EPCAM) as well as butyrophilin subfamily 2 member A1 (BTN2A1), cadherin-4 (CDH4) and endothelin-converting enzyme 1 (ECE1) (**Fig 4A and S3 Table**). To validate PMP results, flow cytometry was performed on HFFs infected with either an HCMV variant encoding a UL32-GFP fusion protein or the same virus additionally deleted for the US1-11 region. Gating on GFP+ HCMV-infected cells confirmed that the US1-11 region was required to downregulate MHC-I, integrin α4 and THBD (**Fig 4B**). In a further test with the specific HCMV ΔUS2 mutant used for PMP, infected cells were distinguished by their downregulation of MHC-I. MHC-I downregulation is unaffected with the HCMV ΔUS2 mutant due to redundancy with the US3/6/11 gene products (**S2 Table**). By gating on MHC-I^lo^ HCMV-infected cells we confirmed that downregulation of integrin α4 and THBD was specifically dependent on US2 (**Fig 4C**). Thus, we confirmed that many of the key US2 targets in THP-1 cells were also downregulated in HFFs in the context of HCMV infection.

### US2 decreases integrin-mediated cell adhesion of differentiated THP-1 cells during lytic HCMV infection

**Fig 5:**
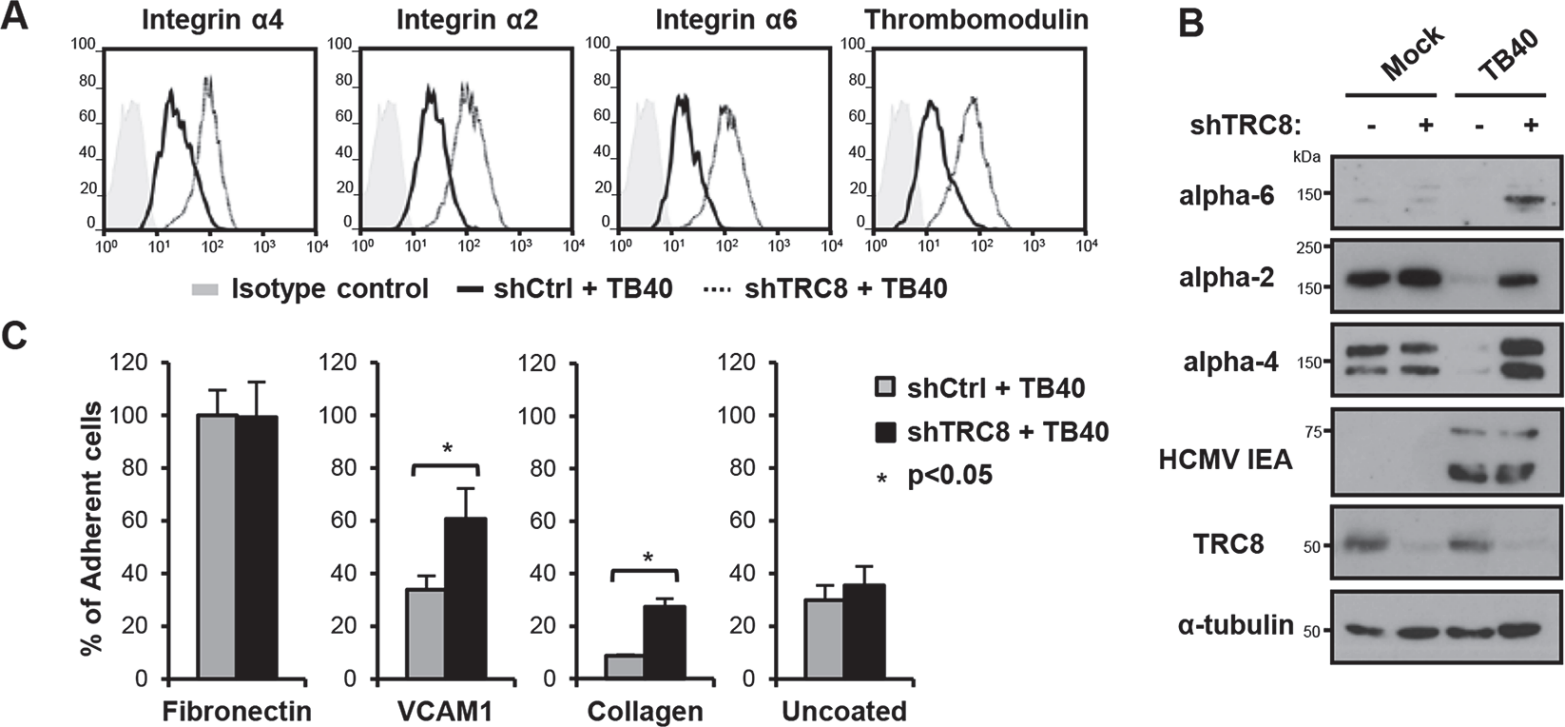
US2/TRC8-mediated degradation of α integrins reduces cell adhesion of lytically HCMV infected THP-1 cells. (**A,B**) TRC8 depletion abolishes US2-induced α integrin degradation in HCMV infected THP-1 cells. THP-1 cells stably expressing a control shRNA (shCtrl) or shRNA targeting TRC8 (shTRC8) were PMA activated and infected with HCMV TB40 UL32-GFP (moi 25). Expression of indicated integrins was assessed at 96 hours post-infection by flow cytometry (**A**) or immunoblotting (**B**). (**C**) TRC8/US2-mediated degradation of α integrins reduces cell adhesion of HCMV-infected THP-1 cells. THP-1 cells were infected as above and at 96 hours post-infection counted, and seeded on tissue culture plates coated with fibronectin, recombinant VCAM-1-Fc, collagen or uncoated. After a 1 hour (fibronectin, VCAM-1, uncoated; n = 4) or 2 hours (collagen n = 2) incubation, loosely attached cells were washed off and the number of adherent cells was determined by CyQUANT-NF assay. Data show adhesion relative to fibronectin binding of control cells as mean ± SEM. p-values were calculated using paired Student’s t-test.

HCMV infected myeloid cells are thought to play a key role in the spreading of virus *in vivo*. Effective mechanisms of immune evasion will be important for enabling viral reactivation in the presence of a primed immune system. We therefore tested whether the US2-induced substrate downregulation observed in whole HCMV-infected HFFs could be replicated in differentiated THP-1 cells, and specifically how US2-induced integrin downregulation affects cell adhesion. PMA-activated THP-1 cells were infected with the endothelial-tropic HCMV strain TB40 harboring a UL32-GFP marker [37]. Since a ΔUS2 mutant of this strain was not available, we inactivated US2 function by a stable TRC8 knockdown, prior to TB40 infection. Comparing control to TRC8 knock-down, we observe a striking US2/TRC8-dependent downregulation (**Fig 5A**) and degradation (**Fig 5B lanes 3 and 4**) of integrins α2, α4, α6 and thrombomodulin in HCMV-infected cells. This downregulation is therefore similar to that observed following HFF infection with the HCMV Merlin strain (Fig 4A, 4B, and 4C). Integrin α6, which is not detected in basal THP-1 cells (**Fig 5B lanes 1 and 2**), was markedly induced upon viral infection and concomitantly downregulated via the US2/TRC8 pathway (**Fig 5B lanes 3 and 4**), suggesting a potent anti-viral role counteracted by US2. A similar upregulation upon virus infection and subsequent downregulation by US2 was observed for thrombomodulin in HFF cells (**Fig 4B**).

To assess the functional consequence of US2-induced integrin downregulation in HCMV infected cells, TB40-infected THP-1 cells were allowed to adhere to fibronectin, recombinant VCAM-1, collagen or uncoated tissue culture wells. TB40-infected THP-1 cells with a control knock-down showed significantly decreased binding to both VCAM-1 and collagen, but not uncoated wells, compared to TRC8 knock-down cells (**Fig 5C**). VCAM-1 and collagen are respectively substrates for the US2-targetted integrins α4 and α2. Binding to fibronectin—a less specific substrate for integrin α4—was unaffected by TRC8 knock-down. This lack of an effect is likely due to up-regulation of integrin αV in PMA-activated THP-1 cells; this β3-associated integrin binds readily to fibronectin but is not a US2 substrate. In conclusion we show α integrins including α2, α4 and α6 are specific US2 substrates that are degraded in a TRC8-dependent manner upon both US2 single gene expression and whole HCMV infection, thereby reducing cell adhesion of myeloid cells to a variety of substrates.

### US2 and UL141 co-operate to downregulate the NK cell ligand CD112

**Fig 6:**
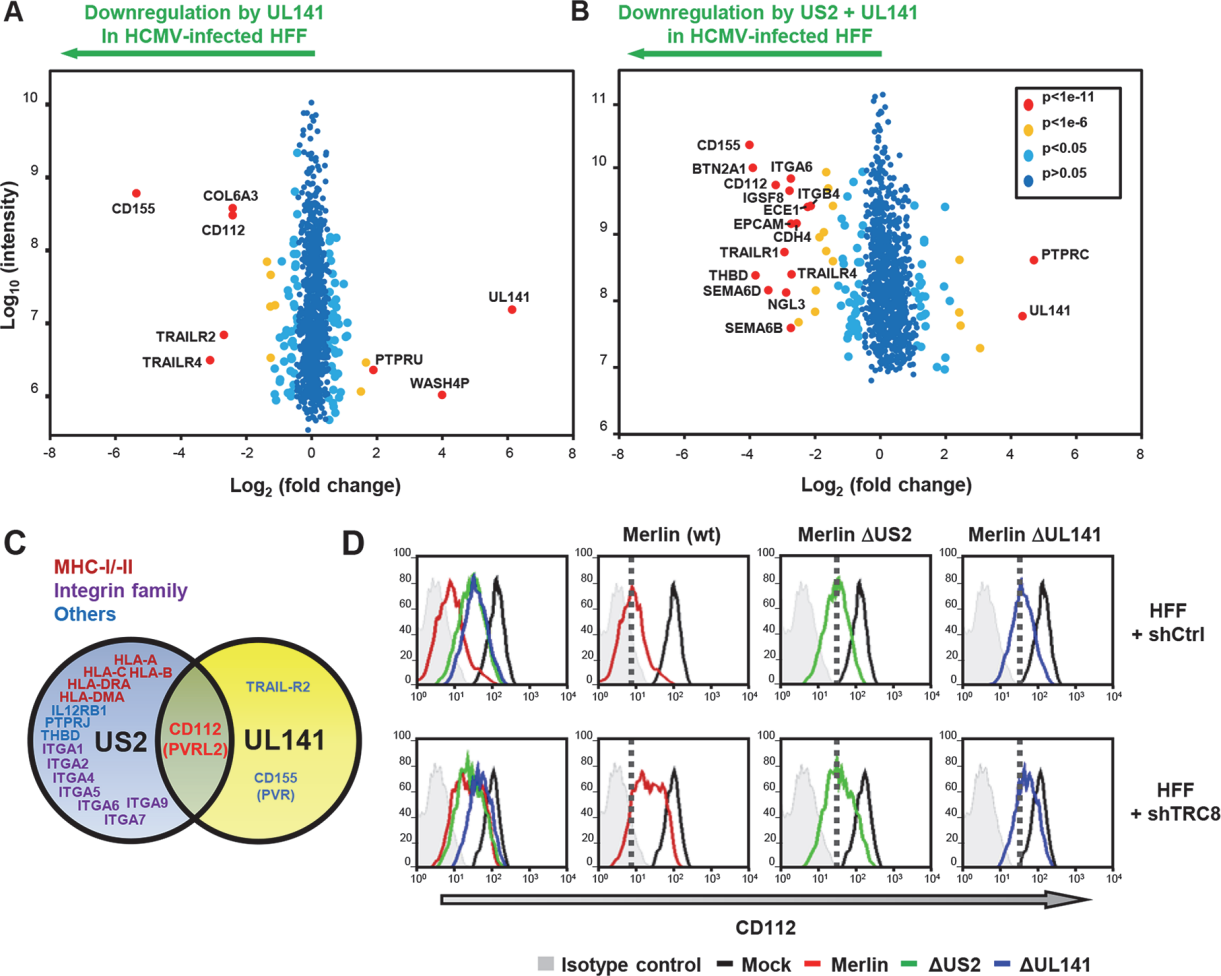
UL141 and US2 are both essential for surface down-regulation of CD112. (**A,B**) Scatter plots of proteins identified in PMP and quantified by 2 or more unique peptides, for HFF infected with (**A**) HCMV wild-type *vs*. ΔUL141 and (**B**) HCMV wild-type vs. a ΔUL141ΔUS2 double deletion mutant. Fold change (wild-type/specific gene deletion HCMV infected human fibroblasts) is shown as log_2_ ratio on the x axis and the summed peptide intensity on the y-axis as log_10_. Proteins unaltered by HCMV UL141 or combined US2/UL141 gene deletion locate to the center of the plots (0-fold change), whereas proteins left or right of center represent respectively proteins down- or up-regulated by HCMV in a UL141 (**A**) or combined US2/UL141-dependent (**B**) manner. Significance B was used to estimate p values. (**C**) Schematic showing verified substrates of US2 and UL141. Substrates identified by PMP and confirmed by us or previous studies are indicated. (**D**) HCMV downregulation of cell surface CD112 requires US2, UL141 and TRC8. Cell surface expression of CD112 was determined in HFF infected with mock or HCMV wild-type, ΔUS2 or ΔUL141 (moi 5) at 72 hours post infection. HFFs were stably transduced with scrambled shRNA (HFF + shCtrl) (top panel) or shTRC8 (HFF + shTRC8) prior to HCMV infection (lower panel). Dotted lines indicate the mean CD112 expression in HFFs with control shRNA infected by indicated virus. See also **S3 Table**.

HCMV UL141 is a powerful NK cell evasion gene that downregulates NK cell ligands CD112, CD155 and the death receptor TRAIL-R2 from the cell surface [25, 26, 38]. Our previous data indicated that UL141 requires an additional unmapped HCMV function to efficiently downregulate CD112 [25]. The PMP study revealed that US2 was also able to alter CD112 expression, both independently and in the context of HCMV infection (**Figs 1A and 4A and S1 Table**), so we further analyzed and compared specific UL141 and US2 deletion mutants. Proteomic analysis of HFF cells infected with a HCMV UL141 deletion mutant versus wild-type HCMV confirmed UL141-dependent down-regulation of the known UL141 targets: CD155, TRAIL-R2 and CD112. TRAILR4 was identified as a novel UL141 target (**Fig 6A**). While expression of the US2 gene alone caused only a modest downregulation of CD112 (2.4 fold) (**Fig 1A and S1 Table**), in the context of whole virus infection, the observed robust CD112 downregulation was clearly dependent on both US2 and UL141 (**Figs 4A and 6A and S3 Table)**. A further PMP experiment using a dual ΔUS2ΔUL141 HCMV deletion mutant (**Fig 6B and S3 Table**) confirmed many of the changes we observed using single gene deletion viruses, and showed that CD112 downregulation was most efficient in the presence of both viral genes (Fig 6A, 6B, and 6C **; S3 Table)**. These results suggest a requirement for both US2 and UL141 for effective CD112 downregulation.

### UL141 retains CD112 in the ER enhancing US2 mediated degradation

UL141 is a predominantly ER-resident viral protein which, in contrast to US2, does not actively promote proteolysis of CD155 or TRAIL-R2 but sequesters them in the ER, thus preventing their further trafficking through the secretory pathway [25, 26, 38]. We hypothesized that UL141 might retain CD112 in the ER, and promote its transfer to US2 for TRC8-dependent ubiquitination and subsequent degradation. Whereas UL141 or US2 alone caused only a partial cell surface down-regulation of CD112 within the viral context (**Fig 6D**; Merlin ΔUS2–3.6x; Merlin ΔUL141–2.8x), their combined action showed a more than additive effect (wt Merlin -13.3x), suggesting synergy between the two immune evasion genes. Cooperativity was also observed between TRC8 and UL141. Cell surface expression of CD112 was partially rescued by TRC8 depletion of cells infected with wild-type HCMV or HCMVΔUL141, but not the HCMVΔUS2 deletion mutant (**Fig 6C**). TRC8-dependent downregulation of CD112 is thus dependent on US2 within the viral context.

**Fig 7:**
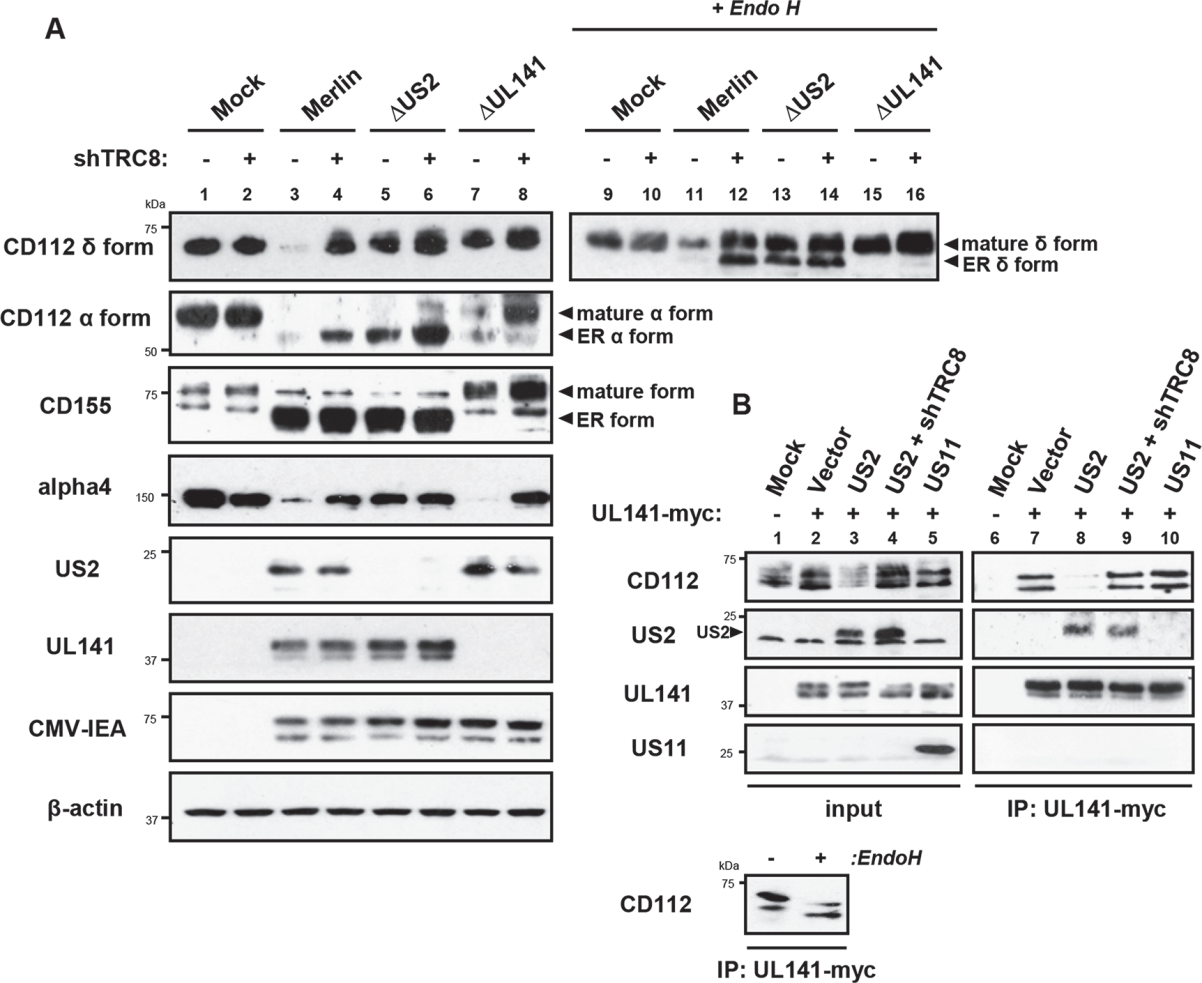
UL141 retains CD112 in the ER and enhances US2 mediated degradation. ER retention of CD112 is mediated by UL141 and only detected upon depletion of US2 or TRC8. (**A**) HFF infected with mock, HCMV wild-type, ΔUS2 or ΔUL141 (moi 5) were analyzed for the indicated proteins at 72 hours post infection, in the presence (shTRC8+) or absence (shTRC8-) of TRC8 depletion. (**B**) UL141 retains CD112 in the ER prior to TRC8-dependent degradation by US2. THP-1 cells stably expressing US2, US11, UL141-Myc or shTRC8 were solubilized in 1% digitonin, immunoprecipitated with anti-Myc antibody and the indicated proteins visualized by immunoblot. See also **S4 Fig**.

Two versions of CD112 are produced by differential splicing; the short α and long δ variants can be distinguished using antibodies specific for their cytosolic tails [39] (**S4 Fig**). Following wild-type HCMV infection, both CD112 α and δ forms were degraded (**Fig 7A, lane 3 *vs*. lane 1**). However, TRC8 depletion, or the absence of US2 (HCMV ΔUS2), rescued the CD112 δ form in both its mature Endo H resistant form (**Fig 7A, CD112 δ blots, lane 11 *vs*. 12 and 11 *vs*. 13, upper bands**) and its immature, ER resident, Endo H sensitive forms (**lower bands**). A similar pattern was seen with the CD112 α isoform, which was degraded by wild-type HCMV and restored specifically in its immature form by a HCMV US2 deletion mutant or TRC8 depletion (**Fig 7A CD112 α blots, lane 3 *vs*. 4 and 3 *vs*. 5)**. The mature form of both the α and δ isoform was only fully restored upon combined UL141 deletion (HCMV ΔUL141) and TRC8 depletion (**Fig 7A lane 3 *vs*. 8 and 11 *vs*. 16**). Therefore, the ability of UL141 to retain both CD112 isoforms in the ER was only revealed in the absence of US2 or following TRC8 depletion which abrogates US2-induced CD112 degradation. In contrast, CD155 downmodulation was solely dependent on UL141 for retention in the ER, and integrin α4 required only US2 in order to be degraded during virus infection (**Fig 7A, CD155 and integrin α4 blots)**.

Together the data indicate that US2 and UL141 co-operate to prevent CD112 cell surface expression. Indeed UL141 and US2 appear capable of interacting, as evidenced by US2 co-immunoprecipitation with UL141 (**Fig 7B, lane 8)**. UL141 also directly associated with its substrate CD112 (**Fig 7B, lane 7**). This interaction is lost in the presence of US2 due to CD112 degradation, but was rescued following TRC8 depletion (**Fig 7B, lanes 8 and 9**). UL141 co-precipitated US2 in both the presence and absence of TRC8 indicating UL141 itself is not degraded by US2 (**Fig 7B, lanes 8 and 9**). The control viral protein US11 is not found in association with UL141. Collectively our results provide a remarkable example of cooperativity between two unrelated viral proteins with diverse functions.

While the retention of CD112 in the ER by UL141 is inefficient and can easily be overcome, teaming up with US2 promoted CD112 degradation via the TRC8-dependent pathway and provides an efficient mechanism of controlling expression of this cellular protein.

## Discussion

**Fig 8:**
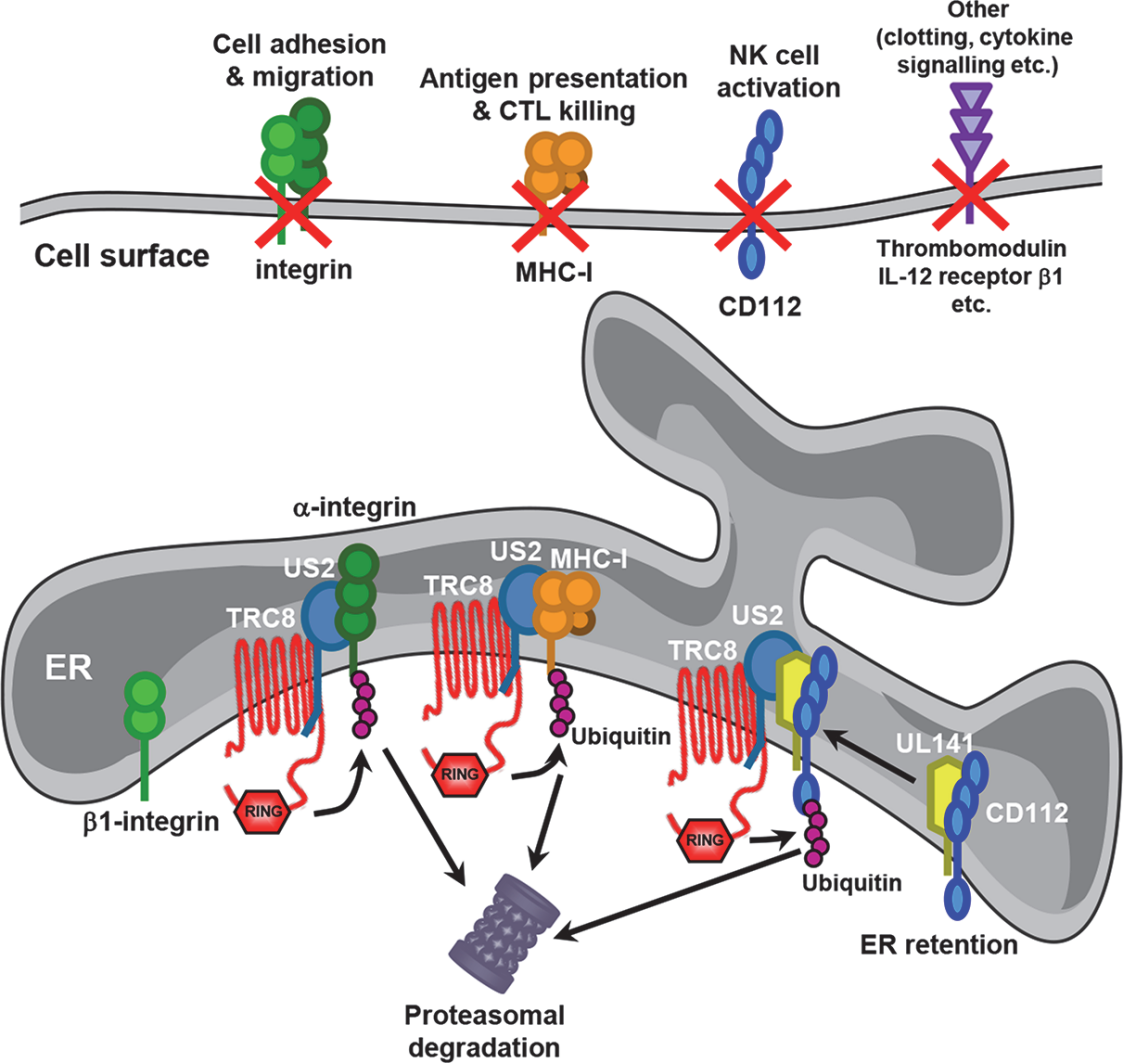
Model for US2 action in HCMV-infected cells. US2 targets a wide variety of novel cell surface substrates, including integrins, the NK cell ligand CD112, the anti-coagulation factor thrombomodulin and the IL-12 receptor β 1. The majority of these targets are directly targeted for proteasomal degradation in a TRC8 E3 ligase dependent pathway. β1 integrin is not itself degraded, but accumulates in an ER-resident immature form following US2-induced degradation of its α integrin interaction partner. CD112 is retained in the ER by UL141 from where US2 promotes the TRC8-dependent degradation. The functional consequences of US2 substrate degradation include diminished cell adhesion, migration, NK cell activation and antigen presentation.

This study demonstrates the power of Plasma Membrane Profiling (PMP) as an unbiased approach to establish a global picture of how individual viral genes modulate the cell surface proteome. Specific antibodies have traditionally been used to determine the changes in cell surface proteins upon viral infection. This straightforward approach has proven particularly useful in tracking changes in expression of critical immune effector cell ligands (e.g. MHC-I) during the course of an infection. By design, this candidate approach is inevitably selective and cannot, therefore, provide a complete picture of the effect of virus-encoded immunomodulatory functions on the cell. By deploying PMP we were able to demonstrate the precision with which US3, US6 and US11 specifically target MHC molecules, whereas US2 was revealed to be a pleotropic modulator of cell surface receptors whose function extends beyond T cell evasion to impact on NK cell function, cell adhesion, signaling and coagulation (**Fig 8**).

We identified many novel cellular substrates that require US2 for their downregulation. For all targets examined, US2-mediated degradation was dependent on the TRC8 E3 ligase, indicating that HCMV-mediated appropriation of this cellular ubiquitin ligase provides a common pathway for the ER-associated degradation of US2 substrates. While US2 alone is sufficient for the downregulation of the majority of new targets, effective removal of the NK cell ligand CD112 requires co-operation between UL141, to retain CD112 in the ER, and US2 to initiate CD112 degradation. The recruitment of US2 by UL141 greatly enhances the efficiency with which CD112 is downregulated and the US2-dependant degradative pathway provides a potential conduit by which host proteins retained in the ER can be targeted for degradation, and may be exploited by other HCMV proteins. US3, like UL141, retains MHC molecules in the ER from where they are degraded by the US2/TRC8 complex [17, 18]. Whereas US3 is an HCMV immediate early gene with expression peaking at 8 hours post-infection [40], UL141 reaches maximum expression at 4–5 days post-infection [26], suggesting US2 might change substrate specificity during the HCMV life cycle. Reliance on different viral retention factors might therefore enhance the flexibility of the US2/TRC8 degradation hub which may therefore be customized towards specific requirements at different stages of the viral life cycle. Antigen presentation may be an acute problem early in viral infection, requiring US3, while NK cell killing, and the requirement for UL141, becomes critical as MHC-I levels on the cell surface decline.

While the US2/TRC8 hub induces degradation of the UL141-substrate CD112, this mechanism is not deployed against UL141’s other targets: CD155 and TRAIL-R2 [26]. Since these three main cellular targets of UL141 are all implicated in distinct intracellular signaling pathways, there may be additional benefit to the virus in retaining CD155 and TRAIL-R2 in an intracellular compartment while targeting CD112 to the proteasome. Alternatively binding to CD155 and/or TRAIL-R2 might be incompatible with US2 binding to UL141. Recent structural analysis suggests the Ig-like domain of UL141 is a structural mimic of TIGIT thus allowing CD155 binding [41], whereas the interaction between UL141 and TRAIL-R2 involves a separate, non-canonical death receptor interaction site. [42]. As it is unusual for an immunomodulator to selectively target multiple unrelated receptors, further understanding of UL141 function may highlight important aspects of the evolution of immune recognition and modulation. It will be of particular interest to gain further structural insight into the CD112-UL141-US2 complex.

The largest group of novel targets downregulated by US2 were the integrins, a large family of 18 α and 8 β chains that assemble into 24 different heterodimers. HCMV-induced integrin down-regulation was first reported for integrin α1β1 [43], and we here show that US2 down-regulates a variety of integrins including α1, α2, α4, α5, α6, α7, β1 and β4. αβ-heterodimer assembly is a prerequisite for integrin maturation and transport to the cell surface [44–46]. Our data suggest that α integrins are targeted by US2 for TRC8-induced ubiquitination and proteasomal degradation, whereas the β1 subunit is retained in the ER due to the absence of its α integrin binding partner. Whether each α chain is individually recognized by US2 and degraded, or US2 binds the shared β1 integrin, which is itself protected, but leads to the degradation of any associated α chain, remains unclear. Our preliminary experiments favour the former scenario, as alpha chains were still degraded in cells depleted of β1 integrin, suggesting that alpha chains are indeed direct targets of US2. However, we cannot exclude that, despite an effective depletion, any remaining β1 integrin, might still target alpha chains for degradation. Furthermore, no common motif in α integrins targeted by US2 has been identified. It is even less clear how US2 recognizes the broad range of substrates (MHC-I, integrins, thrombomodulin, the IL-12 receptor β1 subunit) that share no apparent structural features.

Integrins mediate cellular attachment to a wide range of extracellular proteins, and control multiple cellular functions, including morphology, migration and differentiation [47]. US2-induced α integrin degradation is predicted to have a broad impact on the physiology of HCMV infected cells. In this perspective integrin α6 is of interest as it is induced following HCMV infection and concomitantly downregulated by US2, a pattern reminiscent of antiviral proteins. Indeed, integrin α6 is essential for dendritic cell (DC) migration across the laminin and collagen IV rich basement membranes to reach the draining lymph node for antigen presentation [48, 49]. Integrin α6 specific blocking antibodies inhibit DC migration to lymph nodes [48], and US2-induced integrin degradation should therefore counteract infection-induced DC migration. This might be particularly relevant during viral reactivation from latency when the immune system is already primed to eradicate early stage infection. Additional US2-targetted integrins may also inhibit DC migration: integrin α1 and α2 are receptors for the basement membrane component collagen IV, while integrin α4 and α5 are likely required for DC reverse migration across the endothelial cell layer into the lymph [50]. Furthermore, the α4 integrin is essential for leukocyte transendothelial migration from blood into peripheral tissue and plays a prominent role in immune surveillance [47]. Indeed a monoclonal antibody targeting α4β1 and α4β7 is in clinical use for the treatment of autoimmune diseases, and reduces inflammation by preventing leukocyte extravasation into the tissue [51, 52]. The downregulation of α4β1 by US2 may thus prevent circulating virus-infected myeloid cells from responding to chemoattractants and homing.

Cell migration remains an understudied area of viral immune evasion. A global comparison of plasma membrane proteins altered upon HCMV infection showed that cell surface proteins involved in adhesion and migration are a major target for HCMV [30], and additional HCMV genes likely contribute to HCMV’s modulation of cell migration. In addition to integrins, downregulation of VCAM-1, at least eight protocadherins, five plexins and two ephrins was seen [30]. Furthermore, the actin cytoskeleton of HCMV-infected cells is heavily reorganized by UL135 which hijacks the WAVE2 complex and prevents the formation of focal adhesions [53]. Furthermore, in latent HCMV infection, UL138 likely inhibits DC migration via degradation of the multidrug transporter MRP1 which is essential for leukotriene C4 (LTC4) secretion [29, 54].

Although of potential benefit to viral immune evasion, US2-mediated degradation of cell surface receptors could also potentially contribute to the pathophysiology associated with congenital HCMV infection. Several integrins targeted by US2 (α1, α4, α5, α6 and β1) are required for placentation or fetal development [47]. Integrin α4 deficiency for example is embryonically lethal in mice due to cardiac defects and defective placentation [55]. Depending on the cell types infected in pregnancy, US2’s ability to downregulate integrin family members could contribute to the fetal damage associated with HCMV infection. Indeed, HCMV-induced downregulation of integrin α1β1 has been associated with impaired cytotrophoblast invasion and placentation [56].

The endothelial cell surface protein thrombomodulin (THBD) is another US2 substrate that might contribute to HCMV-associated fetal defects. THBD alters thrombin’s substrate specificity from pro-coagulant and pro-inflammatory to anti-coagulant and anti-inflammatory [57] and its deficiency leads to lethal consumptive coagulopathy in embryonic blood vessel endothelium. Since, the endothelium is a common site of HCMV infection *in vivo*, US2-induced THBD degradation may contribute to both the coagulopathy and severe fetal thrombotic vasculopathy seen in congenital HCMV infection. THBD is also a marker for a human DC subset proficient in antigen cross-presentation, with similarities to mouse CD8+ DCs [58, 59]. While the exact role of THBD in these DCs is unknown, THBD regulates substrate affinity of the TLR4 co-receptor CD14 [60], suggesting a potential broader role in antiviral immunity, which may explain its induction in HCMV-infected cells and concomitant downregulation by US2.

Our study emphasizes the key role of US2 in combating different HCMV host defense pathways through the downregulation of multiple cell surface receptors. As we are particularly interested in HCMV evasion of the cellular immune response, this study focused on plasma membrane proteins. Additional US2 substrates may yet be identified by analysis of ER-resident proteins. Indeed, as shown for UL141, other viral proteins which retain host receptors in the ER may also cooperate with US2 to degrade their cargo through the common pathway of TRC8-dependent degradation.

## Materials and Methods

### Cell culture

THP-1 cells and HFF cells (System Bioscience) were grown in RPMI-1640 or DMEM respectively (PAA), with 10% heat-inactivated fetal bovine serum (FCS; PAA) and penicillin/streptomycin (pen/strep, Sigma).

### HCMV viruses and infections

The HCMV strain Merlin is designated the reference HCMV genome sequence by the National Center for Biotechnology Information [61] and is available as a BAC clone [62]. Merlin BAC derived clone Merlin wild-type used for this study contains point mutations in RL13 and UL128, enhancing replication in fibroblasts [62]. Generation of virus recombinants and stocks was described previously [62]. All recombinants were validated by whole genome Illumina sequencing (**S4 Table**). Merlin ΔUS2 has a deletion of the US2 ORF; Merlin ΔUL141 has deletions of the UL141 ORF; GFP tagged Merlin ΔUL16,18 ΔUS1-11 has deletions of the UL16, UL18, US1-11 ORFs and contains a UL32-GFP fusion; GFP tagged Merlin ΔUL16,18 (Merlin delta UL16/UL18, UL32-GFP) was described previously [25]. HFF cells were infected with HCMV at indicated MOI for 72h.

The GFP-tagged endothelial-tropic HCMV TB40 strain was originally created by Christian Sinzger (University of Ulm, Germany) [37] and a kind gift from Mark Wills (University of Cambridge, UK). It contains an intact US1-11 region and a UL32-GFP fusion. For THP-1 infections, THP-1 cells were starved for 12-16h in RPMI with 2% FCS, activated for 48h with 100ng/ml PMA (Sigma) and infected with TB40 at MOI 25. This resulted in infection of >95% of cells as estimated by GFP positivity and MHC-I down-regulation. Infected cells were harvested at 96h post-infection.

### Plasmids, lentivirus propagation and transduction

A C-terminal myc-tagged UL141 (HCMV Merlin strain) and HA-tagged human integrin α4 were cloned into the pHRSIN lentivirus vector with a hygromycin B selection cassette. Untagged US2, US2 with a deletion of the C-terminal cytoplasmic tail (aa1-186; US2ΔC'), US3, US6 and US11 were cloned into a pHRSIN lentivirus vector with an IRES CFP and puromycin selection cassette. N-terminal HA-tagged US2 (HA-US2) was cloned into pHRSIN with a puromycin cassette only. The pHRSIN lentivirus expression system was used as described previously [63].

For shRNA-mediated knockdown of TRC8 and integrin β1 expression, hairpin oligonucleotides were designed as described [13, 64], annealed, cloned into the pHR-SIREN lentiviral vector (a gift from Greg Towers, UCL, London). Lentivirus was produced as previously described in 293ET cells and used to transduce THP-1 cells.

### Antibodies and reagents

Primary antibodies used for flow cytometry were: mouse α-conformational MHC-I (W6/32), mouse α-conformational HLA-A2 (BB7.2), mouse α-integrin β1 (Biolegend), mouse α-integrin α1, (BD), mouse α-integrin α2, (BD), mouse α-integrin α4 (BD), rat α-integrin α6 (Biolegend), rabbit α-integrin αV (Santa Cruz), mouse α-thrombomodulin (BD), mouse α-PTPRJ (Medical & Biological Laboratories), mouse α-IL12 receptor β1 (BD), mouse α-CD112 (Santa Cruz), mouse α-CD155 (Abcam) and FITC-conjugated mouse α-transferrin receptor (CD71; BD). AlexaFluor 647 conjugated goat anti-mouse (Life Biosciences) was used as a secondary antibody. Antibodies used for immunoblotting were: mouse α-MHC-I (HC10), rabbit α-calreticulin (Thermo), mouse α-β-actin (Sigma), mouse α-HA (Sigma), rabbit α-integrin α 4, α 5, β1, β3 (Integrin antibody sampler kit; Cell Signaling), rabbit α-Integrin α6 (Cell Signaling), goat α-CD112 (R&D Systems), rabbit α- CD112 δ form (Abcam), rabbit α-CD112 α form (LifeSpan Biosciences), mouse α-CD155 [26], mouse α-HCMV IE antigen (Argene), mouse α-HCMV US2 (a kind gift from Jack R. Bennink, NIH, US), rabbit α-HCMV US11 (a kind gift from Emmanuel Wiertz, University of Utrecht, Netherlands), mouse α-HCMV UL141 [26], mouse α-paxillin (BD) and rabbit α-phospho-paxillin Tyr118 (Cell Signaling). Antibodies used for immune precipitation were: mouse α–HA and α-myc agarose affinity gel (Sigma), mouse α-integrin β1 (Biolegend) and mouse α-ubiquitin (FK1; Millipore) in combination with protein A or protein G-conjugated sepharose (Sigma).

### siRNA

For siRNA gene depletion, cells were transfected using Oligofectamine (Invitrogen) at a final concentration of 40 nM and harvested at 96 h post transfection. The following siRNA oligonucleotides were used (Dharmacon, ON-TARGET PLUS):

CD112 siRNA-1, 5’-GCGCUGAGCAGGUCAUCUU-3’;

CD112 siRNA-2, 5’-GCAUGAGAGCUUCGAGGAA-3’.

### Immunoblotting and immunoprecipitation

For immunoblots, cells were lysed in TBS (pH7.4) with 1% NP40, 0.1% SDS, 5mM IAA, 0.5mM PMSF (Sigma) and 1X complete protease inhibitor (Roche) for 30 min on ice. For immunoprecipitations, cells were lysed in 1% digitonin (Calbiochem) in TBS with 5mM IAA, 0.5mM PMSF (Sigma) and 1X complete protease inhibitor (Roche) for 30 min on ice. Lysates were cleared of cellular debris by centrifugation and pre-cleared using IgG-sepharose (GE Healthcare). Individual proteins were immunoprecipitated using indicated antibodies in combination with Protein A or G sepharose (Sigma), washed extensively and eluted in SDS reducing sample buffer. All samples were heated for 10 min at 50°C, separated by SDS/PAGE and transferred to PVDF membranes (Millipore). Membranes were probed with the indicated antibodies, and reactive bands were visualized with Supersignal West Pico or West Dura (Thermo Fisher Scientific).

### Metabolic labeling and pulse–chase

Cells were starved for 20 min in methionine-free, cysteine-free medium (Sigma), labeled with [^35^S]methionine/[^35^S]cysteine (Amersham) for the indicated time and then chased in medium containing an excess of cold methionine and cysteine (Sigma) at 37°C. Samples taken at the indicated time points were lysed in 1% Triton X-100/TBS with 5mM IAA, 0.5mM PMSF (Sigma) and 1X complete protease inhibitor (Roche) for 30 min on ice. Immunoprecipitations were performed as above.

For visualization of ubiquitinated integrin α4, primary α-HA immune precipitations from ^35^S-labelled cells were eluted at 50oC for 15min in 50μl TBS, containing 1% SDS. Eluates were taken off the beads and after addition of 20μM DTT fully denatured at 70oC for 10min to dissociate interacting proteins. SDS was quenched by the addition of 1ml 1% Triton X-100/TBS with IAA/PMSF/protease inhibitor followed by secondary α-HA or α-ubiquitin immune precipitation.

### Cell adhesion and migration assays

For cell adhesion assays, 96-well tissue culture plates were coated with 20 μg/ml fibronectin (Sigma), 20 μg/ml recombinant VCAM1/Fc (R&D Biosystems) or 200ug/ml collagen (Sigma) in PBS, for 16 h at 4°C and blocked with 0.5% bovine serum albumin (BSA) in PBS for 2 h at 37°C to block nonspecific binding. THP-1 cells expressing single HCMV genes were starved 3 h in serum free RPMI prior to assays, seeded on fibronectin-coated and uncoated plates for 1 h at 37°C and washed 3 times with PBS. Adherent cells numbers were quantified by CyQUANT-NF (Invitrogen) according to the manufacturer’s protocol.

HCMV TB40 infected THP-1 cells were harvested at 96h post-infection using enzyme-free cell dissociation buffer (Life Biosciences), counted and re-seeded on fibronectin, VCAM1, collagen or uncoated plates. Following 1 h (fibronectin, VCAM1, uncoated) or 2 h incubation at 37°C, wells were washed 5 times with PBS, containing 0.5% BSA. Adherent cell numbers were quantified as above.

For cell migration assays, Costar Transwell 24-well plates with 8 μm pore size (Thermo) were coated with 20 μg/ml fibronectin (Sigma) in PBS, for 16 h at 4°C. Cell migration was performed in RPMI with or without the chemotaxis agent MCP-1 (Sigma) at 10 ng/ml and 10% FBS in the lower chamber. After 6 h incubation at 37°C, cells migrated to the lower chamber were counted using a Neubauer counting chamber. All cell adhesion and migration assays were performed in triplicate and p-values were calculated using paired Student’s t-test based on independent experiments.

### Plasma membrane profiling

Plasma membrane profiling was performed as described previously for THP-1 cells, with minor modifications for adherent HFFs [28, 29]. Briefly, for THP-1 cells, 1.5 × 10^8^ of each SILAC-labeled cell type were pooled in a 1:1 ratio. Labeling was as follows: (first experiment, **Fig 1A, top left and bottom right panel**) THP-control (light label); THP-US2 (medium label); THP-US11 (heavy label). (Second experiment, **Fig 1A, top right and bottom left panel**) THP-US6 (light label); THP-control (medium label); THP-US3 (heavy label). Surface sialic acid residues were oxidized with sodium meta-periodate (Thermo) then biotinylated with aminooxy-biotin (Biotium). The reaction was quenched, and the biotinylated cells incubated in a 1% Triton X-100 lysis buffer. Biotinylated glycoproteins were enriched with high affinity streptavidin agarose beads (Pierce) and washed extensively. Captured protein was denatured with DTT, alkylated with iodoacetamide (IAA, Sigma) and digested with trypsin (Promega) on-bead overnight. Tryptic peptides were collected and fractionated (described below). Glycopeptides were eluted using PNGase (New England Biolabs) then desalted using StageTips [65].

For HFF cells, one 150cm^2^ flask of HCMV-infected HFFFs per condition was washed twice with ice-cold PBS. Labeling was as follows: (first experiment, **Fig 4A**) HFF/Merlin-wt (light label), HFF-Merlin ΔUS2 (medium label). (Second experiment, **Fig 6A**) HFF/Merlin-wt (medium label), HFF-Merlin ΔUL141 (heavy label). (third experiment, **Fig 6B**) HFF/Merlin-wt (light label), HFF-Merlin ΔUL141ΔUS2 (medium label). Sialic acid residues were oxidized with sodium meta-periodate (Thermo) then biotinylated with aminooxy-biotin (Biotium). The reaction was quenched, and the biotinylated cells scraped into 1% Triton X-100 lysis buffer. Biotinylated glycoproteins were enriched and digested as described above.

### High pH reverse-phase high pressure liquid chromatography (HpRP-HPLC) fractionation and mass spectrometric analysis

HpRP-HPLC was performed on tryptic peptides as described previously [28]. 90% of each tryptic peptide sample was subjected to HpRP-HPLC fractionation using a Dionex Ultimate 3000 powered by an ICS-3000 SP pump with an Agilent ZORBAX Extend-C18 column (4.6 mm x 250 mm, 5 μm particle size). Peptides were resolved using a linear 40 min 0.1%-40% acetonitrile gradient at pH 10.5. Eluting peptides were collected in 15s fractions. For THP-1 experiments, a total of 30 combined fractions were generated then dried using an Eppendorf Concentrator for LC-MSMS using a NanoAcquity uPLC (Waters, MA, USA) coupled to an LTQ-OrbiTrap XL (Thermo, FL, UA). MS data was acquired between 300 and 2000 m/z at 60,000 fwhm with CID spectra acquired in the LTQ with MSMS switching operating in a top 6 DDA fashion. Fractionated HFF experiments were re-combined to give either 40 or 10 fractions. The 40 fraction experiments were acquired using the OrbiTrap XL as above. The 10 fraction experiments were acquired using a Q Exactive (Thermo) coupled to a RSLC nano3000 (Thermo) with MS data acquired between 400 and 1650 m/z at 75,000 fwhm with HCD fragment spectra acquired in a top 10 DDA fashion.

### Database searching and data processing

Raw MS files were processed using MaxQuant version 1.3.0.5. [66, 67]. Data were searched against concatenated Uniprot human and HCMV databases, and common contaminants [67]. Fragment ion tolerance was set to 0.5 Da with a maximum of 2 missed tryptic cleavage sites. Carbamidomethyl cysteine was defined as a fixed modification, oxidised methionine, N-terminal acetylation and deamidation (NQ) were selected as variable modifications. Reversed decoy databases were used and the false discovery rate for both peptides and proteins were set at 0.01. Protein quantitation utilised razor and unique peptides and required a minimum of 2 ratio counts and normalized protein ratios reported. Peptide re-quantify was enabled in all analyses. Summed intensity represents the sum of all heavy and light labelled peptide intensities for a given protein and was calculated by MaxQuant [68]. Significance B values were calculated and Gene Ontology Cellular Compartment (GOCC) terms added using Perseus version 1.2.0.16 (downloaded from http://maxquant.org). Significance B identifies the significance of outlier protein ratios from the distribution of all ratios with a greater significance given to proteins with a high intensity [68]. Ratios were excluded for proteins identified by <2 unique peptides, or with a variability >150%. We assessed the number of PM proteins identified as described previously [28].

## Supporting Information

S1 Fig US2 but not US11 downregulates integrins α1, α 2, α 4, and β1, thrombomodulin, PTPRJ and IL12 receptor β1, related to Fig 1. Cytofluorometric analysis of THP-1 cells, transduced as indicated. Staining for transferrin receptor and integrin αV was included to show the specificity of US2-induced immune receptor down-regulation.
(TIF)

S2 Fig TRC8 knock-down rescues US2-induced integrin degradation, related to Fig 1. THP-1 cells stably expressing US2 in combination with a control shRNA (shCtrl) or shRNA against TRC8 (shTRC8) were analyzed by immunoblot with the indicated antibodies.
(TIF)

S3 Fig US2 also downregulates integrin α4 and α6 in HFF cells in a TRC8 dependent manner, related to Fig 2. HFF cells stably expressing an empty vector, US2, or US2 with a shRNA against TRC8 (shTRC8) were analyzed by immunoblot with the indicated antibodies.
(TIF)

S4 Fig CD112, α (short form) and δ (long form), could be distinguished by variant specific antibodies, related to Fig 6. HFF cells transfected with two different siRNA against CD112 were analyzed by immunoblot with the indicated antibodies.
(TIF)

S1 Table Cell surface proteome changes in THP-1 cells stably expressing US2 or US11, compared to control cells, related to Fig 1. (XLSX)

S2 Table US2 downregulates cell surface integrins and MHC-I. (XLSX)

S3 Table Cell surface proteome changes in HFF infected with wild-type or deletion strains of HCMV, related to Figs 4 and 5. (XLSX)

S4 Table Gene bank entries of HCMV whole genome-sequencing and primers used to create deletion viruses. (DOCX)
