# Supplementary figures and images for: Plasma Membrane Profiling Defines an Expanded Class of Cell Surface Proteins Selectively Targeted for Degradation by HCMV US2 in Cooperation with UL141
Source: PLoS Pathog. 2015 Apr 14;11(4):e1004811. doi: 10.1371/journal.ppat.1004811 (PMC4397069; doi:10.1371/journal.ppat.1004811)

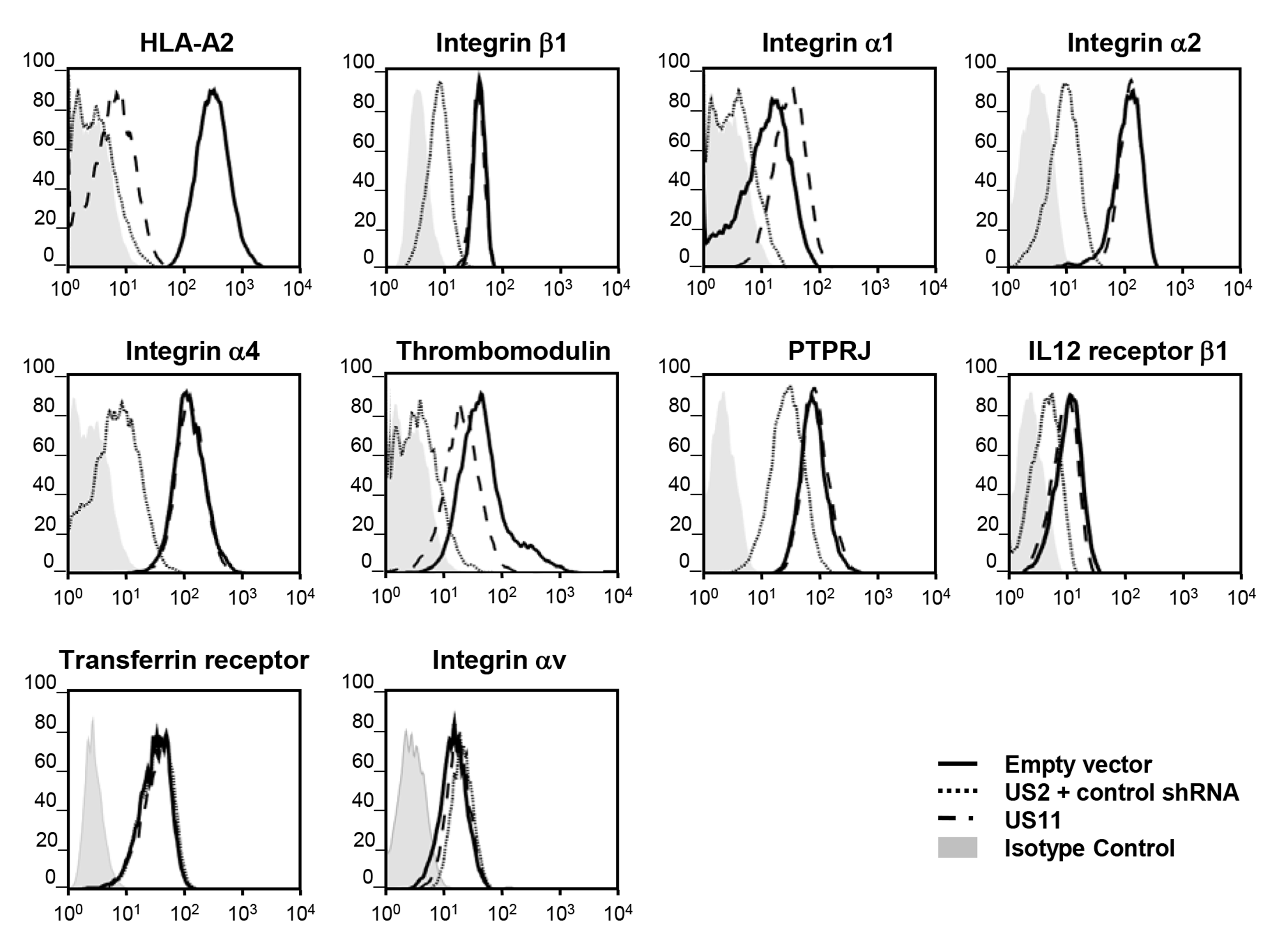

Supplement: S1 Fig — Cytofluorometric analysis of THP-1 cells, transduced as indicated. Staining for transferrin receptor and integrin αV was included to show the specificity of US2-induced immune receptor down-regulation. (TIF) [file ppat.1004811.s001.tif]

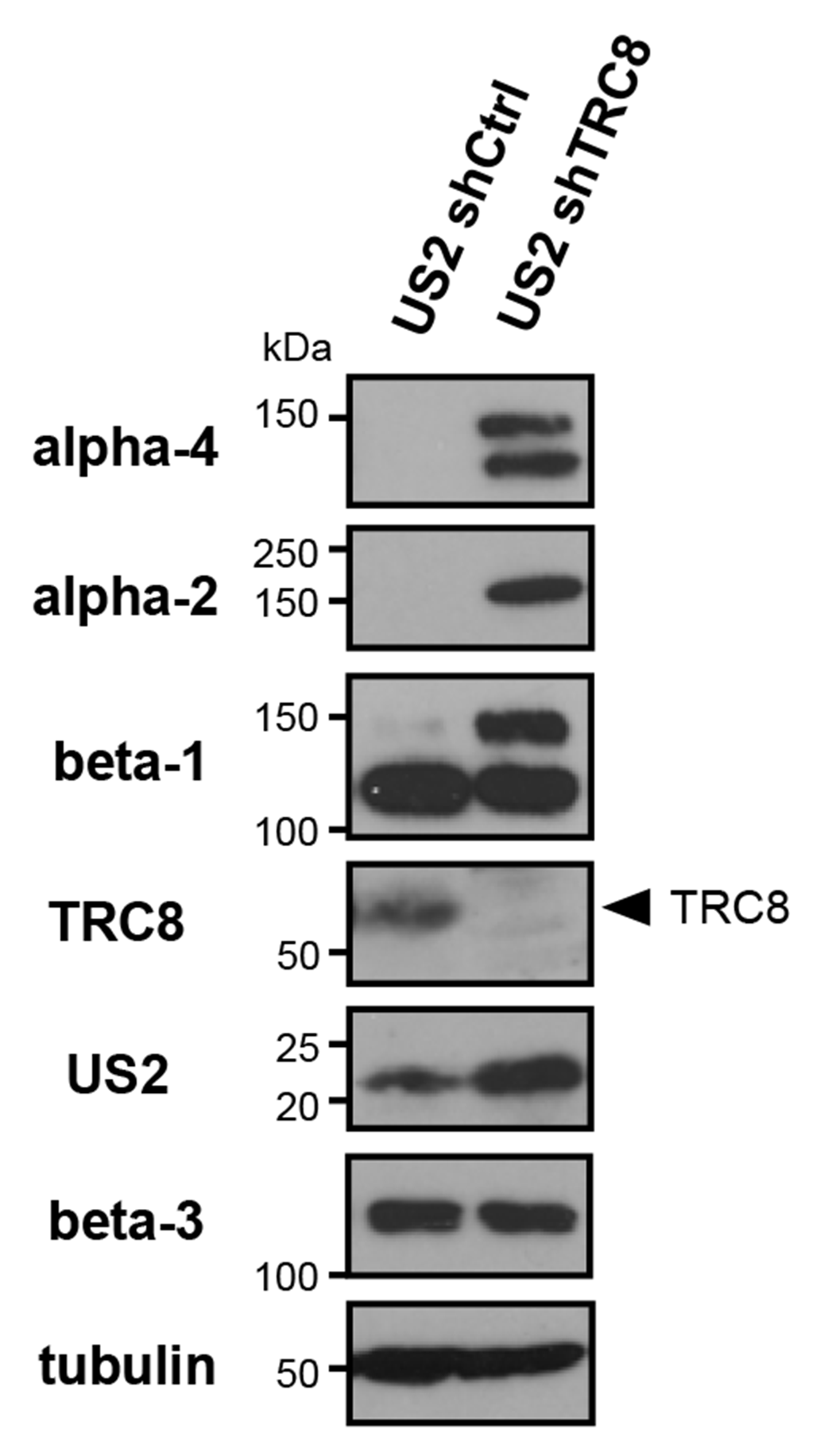

Supplement: S2 Fig — THP-1 cells stably expressing US2 in combination with a control shRNA (shCtrl) or shRNA against TRC8 (shTRC8) were analyzed by immunoblot with the indicated antibodies. (TIF) [file ppat.1004811.s002.tif]

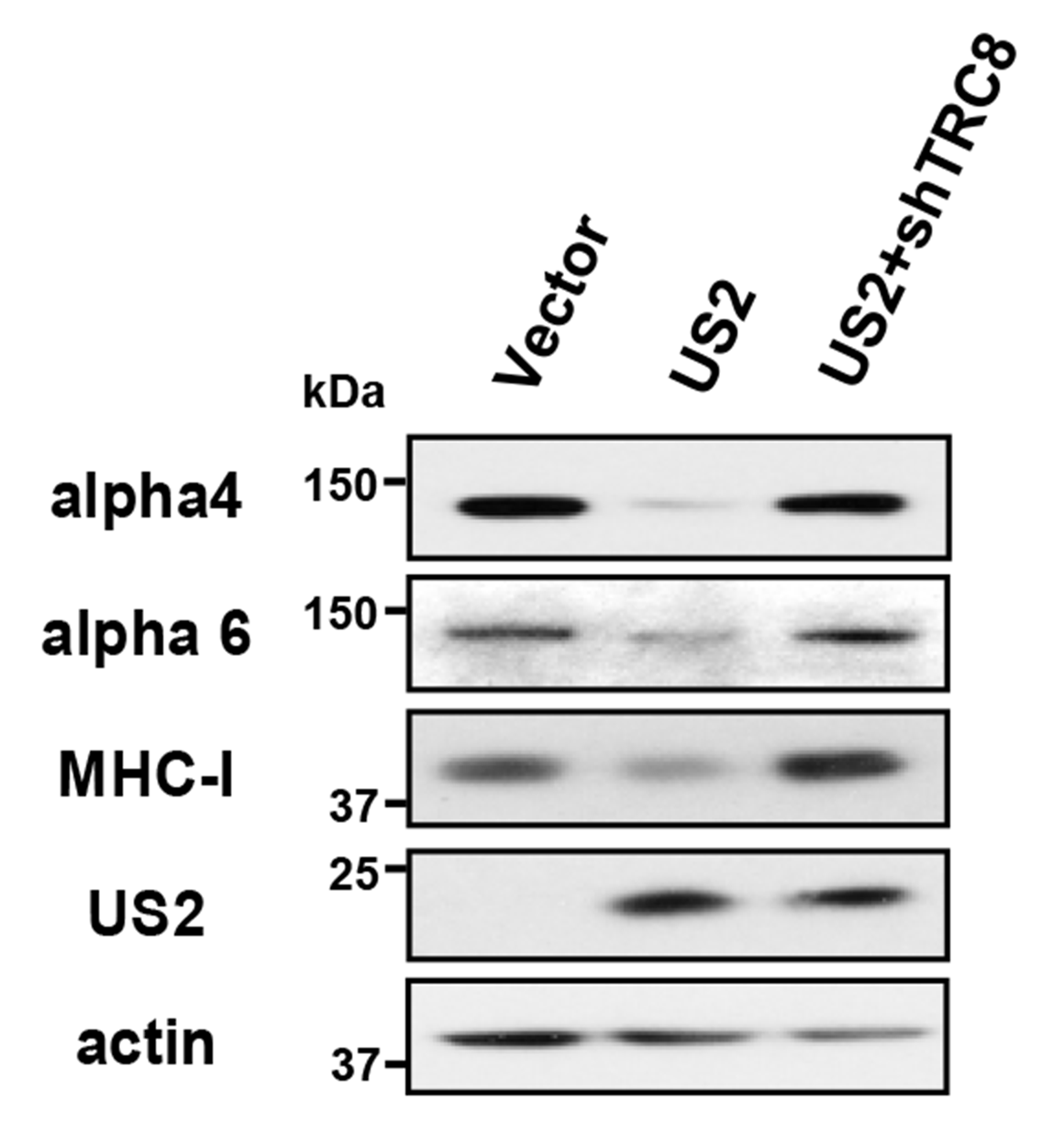

Supplement: S3 Fig — HFF cells stably expressing an empty vector, US2, or US2 with a shRNA against TRC8 (shTRC8) were analyzed by immunoblot with the indicated antibodies. (TIF) [file ppat.1004811.s003.tif]

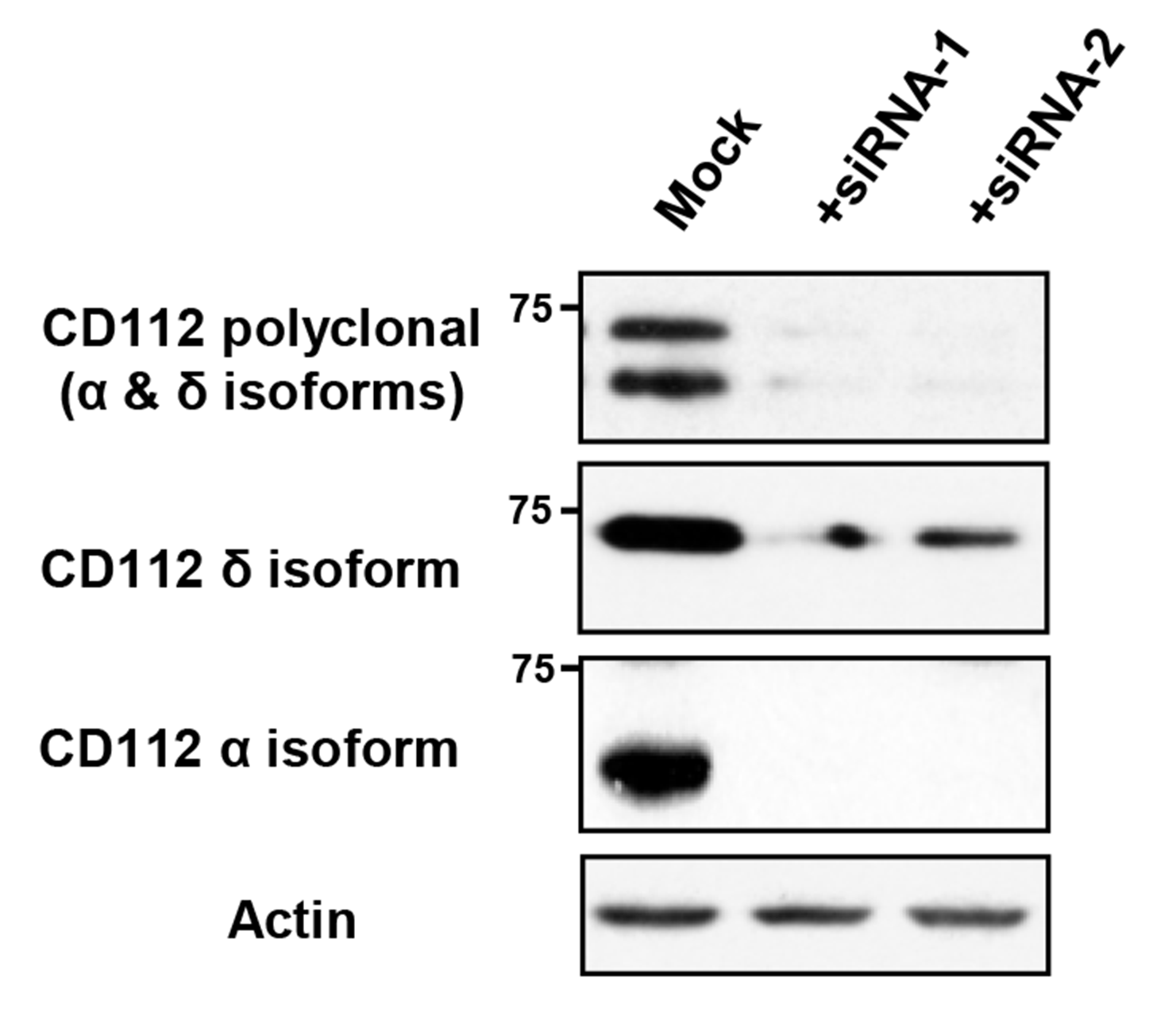

Supplement: S4 Fig — HFF cells transfected with two different siRNA against CD112 were analyzed by immunoblot with the indicated antibodies. (TIF) [file ppat.1004811.s004.tif]
