# Supplementary material for: Plasma Membrane Profiling Defines an Expanded Class of Cell Surface Proteins Selectively Targeted for Degradation by HCMV US2 in Cooperation with UL141
Source: PLoS Pathog. 2015 Apr 14;11(4):e1004811. doi: 10.1371/journal.ppat.1004811 (PMC4397069; doi:10.1371/journal.ppat.1004811)
Supplement: S4 Table — (DOCX) [file ppat.1004811.s008.docx]

| **HCMV recombinant** | **Deletion** | **GenBank Accession Number** | **Reference** |
| --- | --- | --- | --- |
| RCMV1677  (Merlin ΔUS2) | 600nt deletion removing entire US2 | KM192301 | N. A. |
| RCMV1149  (Merlin ΔUL141) | 816 deletion removing entire UL141 | KM192299 | Prod’Homme (2010), Journal of General Virology 91:2034 |

The RCMVs (passage 2) have been whole genome sequenced and deposited to GenBank

Primers used for deletion of the above:

**US2 sacB forward:**

AAT AAA CGA AAC CGA CAT GAC ATA CGT AAT GGG TGC TCG TGG CTA CAT TTA TTG AAA CAA ACC GCG ATC CCG GGC GTC CCT GTG ACG GAA GAT CAC TTC G

**US2 sacB reverse:**

CCT GTT GGT ATT GTT TAT CGT GTA TGT GAC TGT GGA CTG TAA CTT GTC TAT GAT GTG GAT GCG GTT TTT CGT GTG CTA ACT GAG GTT CTT ATG GCT CTT G

**Remove US2:**

ATG GGT GCT CGT GGC TAC ATT TAT TGA AAC AAA CCG CGA TCC CGG GCG TCG GTG AAA CAG CGT GTG GAC TGT ACG CTC TTT CCC AAG TTA TAT CCC AGA G

**UL141 sacB forward:**

caggtagcataggaaacatacggtgaaaatactccaaaatcccaaaaatgccgcgattccccgagtggcccagggagacctgtgacggaagatcacttcg

**UL141 sacB reverse:**

Ccgacgtttgagcggccgacacacggagcaggaacaggcgggcagcgtctctgcgaaaaagggaagaaaagaatcatcctgaggttcttatggctcttg

**Remove UL141:**

atactccaaaatcccaaaaatgccgcgattccccgagtggcccagggagagatgattcttttcttccctttttcgcagagacgctgcccgcctgttcctg
